# Spontaneous Pneumomediastinum in the Postpartum Period: The Macklin Effect

**DOI:** 10.7759/cureus.97232

**Published:** 2025-11-19

**Authors:** Souad Jellam, Mohamed Ijim, Oussama Fikri, Lamyae Amro

**Affiliations:** 1 Pulmonology Department, Centre Hospitalo-Universitaire (CHU) Mohammed VI, Arrazi Hospital, Marrakech, MAR

**Keywords:** dyspnea, macklin effect, pneumomediastinum, postpartum, retrosternal pain

## Abstract

Spontaneous pneumomediastinum (SPM) in the postpartum period is defined by the presence of air within the mediastinum in the absence of any apparent traumatic or iatrogenic cause. We report the case of a 32-year-old woman who developed SPM in the immediate postpartum period. Her medical history was notable only for a previous miscarriage; she had no personal or family history of asthma, atopy, or other respiratory disease. Clinically, the patient presented with acute retrosternal chest pain and sudden-onset dyspnea, accompanied by a single episode of mild hemoptysis following prolonged labor. Physical examination revealed precordial crackles synchronized with the heartbeat (Hamman’s sign). Blood tests showed elevated D-dimer levels. A CT scan confirmed the diagnosis of pneumomediastinum. The patient received conservative management with low-flow oxygen therapy and close clinical and imaging monitoring, resulting in gradual resolution of symptoms and a favorable outcome.

## Introduction

Spontaneous pneumomediastinum (SPM) is defined as the presence of air within the mediastinum in the absence of any evident traumatic or iatrogenic cause [1]. It is a rare, usually benign condition that typically affects young adults following episodes of physical exertion against a closed glottis. Among various situations in this context, the postpartum period after vaginal delivery has occasionally been reported, although it remains extremely rare, with only a few cases described in the literature [2].

This phenomenon is explained by the rupture of alveoli secondary to increased intrathoracic pressure during intense pushing efforts, leading to the dissection of air along the bronchovascular sheaths and its subsequent migration into the mediastinum - a process known as the Macklin effect [3]. The diagnosis is confirmed by radiological imaging, particularly a chest CT scan, which also helps exclude more complicated differential diagnoses such as pulmonary embolism, given the nonspecific symptoms and clinical context. Although a plain chest X-ray may demonstrate mediastinal air, it can miss small volume. Here, we present a case of spontaneous pneumomediastinum occurring in the postpartum period. This case highlights the importance of recognizing postpartum SPM to avoid unnecessary invasive investigations and to raise awareness among clinicians of this rare but self-limiting entity.

## Case presentation

Our patient was a 32-year-old primipara who delivered vaginally at term. Her medical history was notable only for a previous miscarriage, with no personal or family history of atopy or asthma. During labor, she experienced a prolonged course associated with intense and sustained expulsive efforts, managed with epidural analgesia, resulting in the delivery of a healthy neonate. Six hours postpartum, she developed acute retrosternal chest pain and sudden-onset dyspnea, accompanied by a single episode of mild hemoptysis. These symptoms occurred in the context of apyrexia and a generally stable condition. On clinical examination, the patient was tachypneic with a respiratory rate of 30 breaths per minute and tachycardic at 115 beats per minute, with normal blood pressure (120/60 mmHg) and oxygen saturation at 95%. Homans’ sign was negative, and calf palpation was unremarkable. No subcutaneous emphysema was detected. Pleuropulmonary auscultation revealed fine precordial crackles synchronized with the heartbeat, consistent with Hamman’s sign. Blood tests showed elevated D-dimer levels (6500 ng/ml), with otherwise normal results (Table 1).

**Table 1:** Laboratory test results with reference ranges Laboratory test results of the patient showed elevated D-dimer levels.

Parameter	Patient Value	Reference Range
Hemoglobin (g/dl)	13.5	12-16
Platelets (/mm³)	250,000	150,000-400,000
White blood cells (/mm³)	7500	4000-10,000
D-dimer (ng/ml)	6500	<500
C-reactive protein (CRP, mg/l)	5	<10

**Figure 1:**
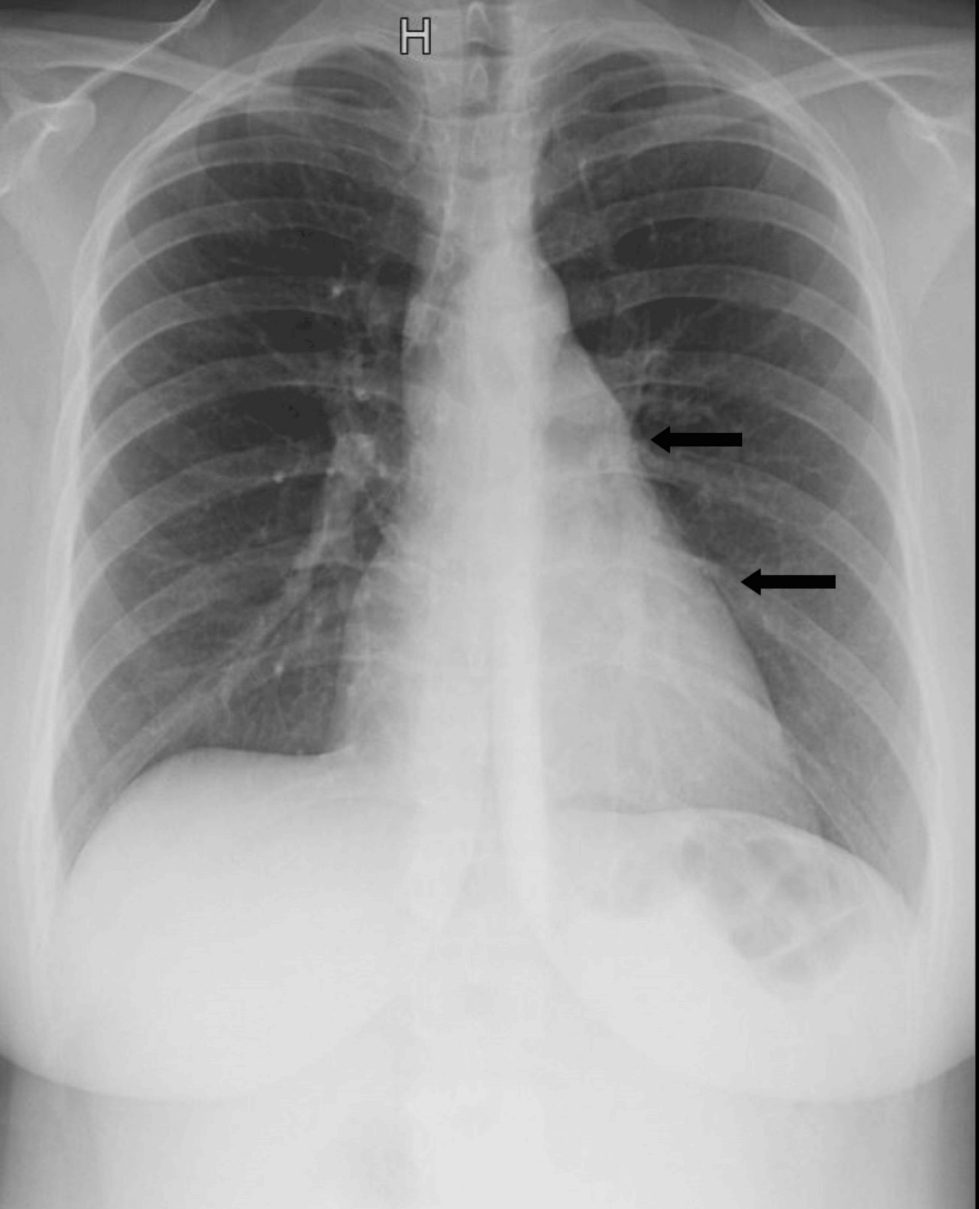
Chest X-ray with the typical appearance of mediastinal pneumothorax A thin, clear border is seen surrounding the cardiac silhouette and thoracic distension, consistent with air accumulation in the mediastinum.

 Chest X-ray showed a fine aeric border surrounding the cardiac silhouette and mild thoracic distension (Figure 1).

**Figure 2:**
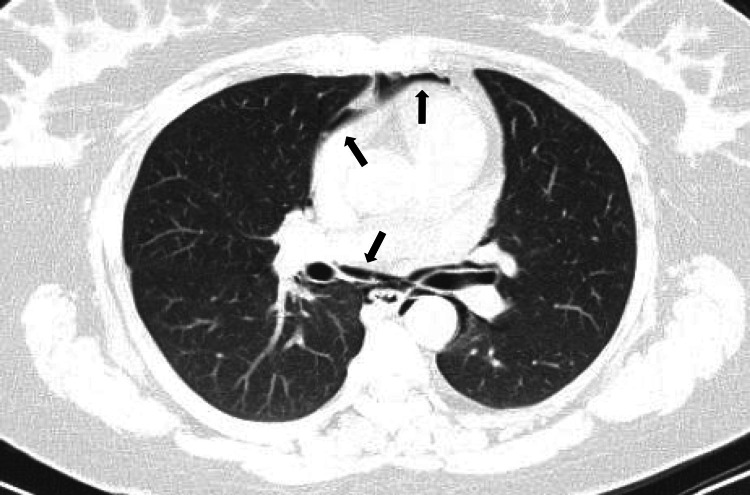
Transverse chest CT scan showing moderate pneumomediastinum (arrows)

**Figure 3:**
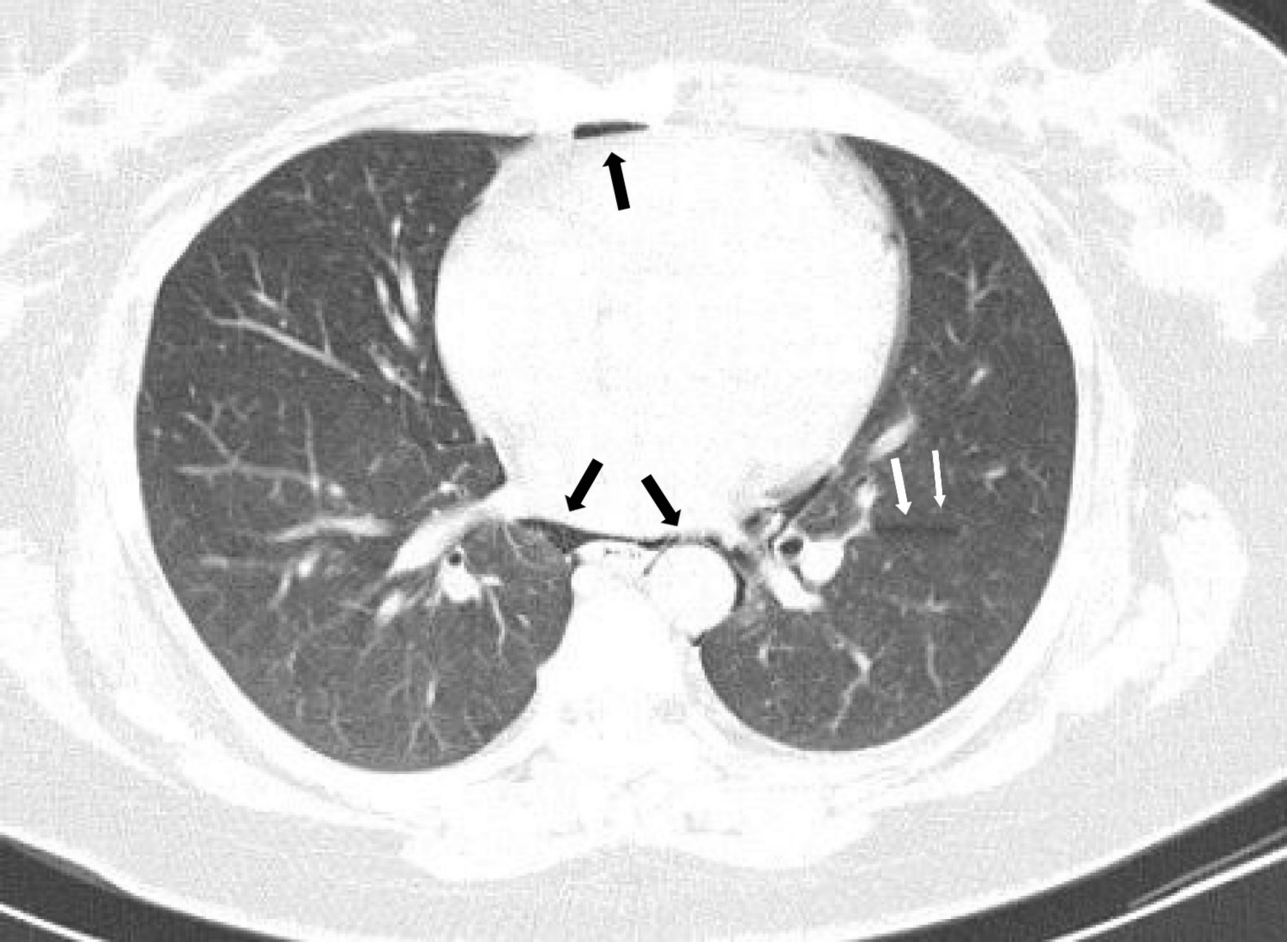
Chest CT scan (transverse section) showing pneumomediastinum due to the Macklin effect The chest CT scan confirmed pneumomediastinum (black arrows) secondary to the Macklin effect, associated with interstitial emphysema dissecting along the peribronchovascular sheaths (white arrows).

**Figure 4:**
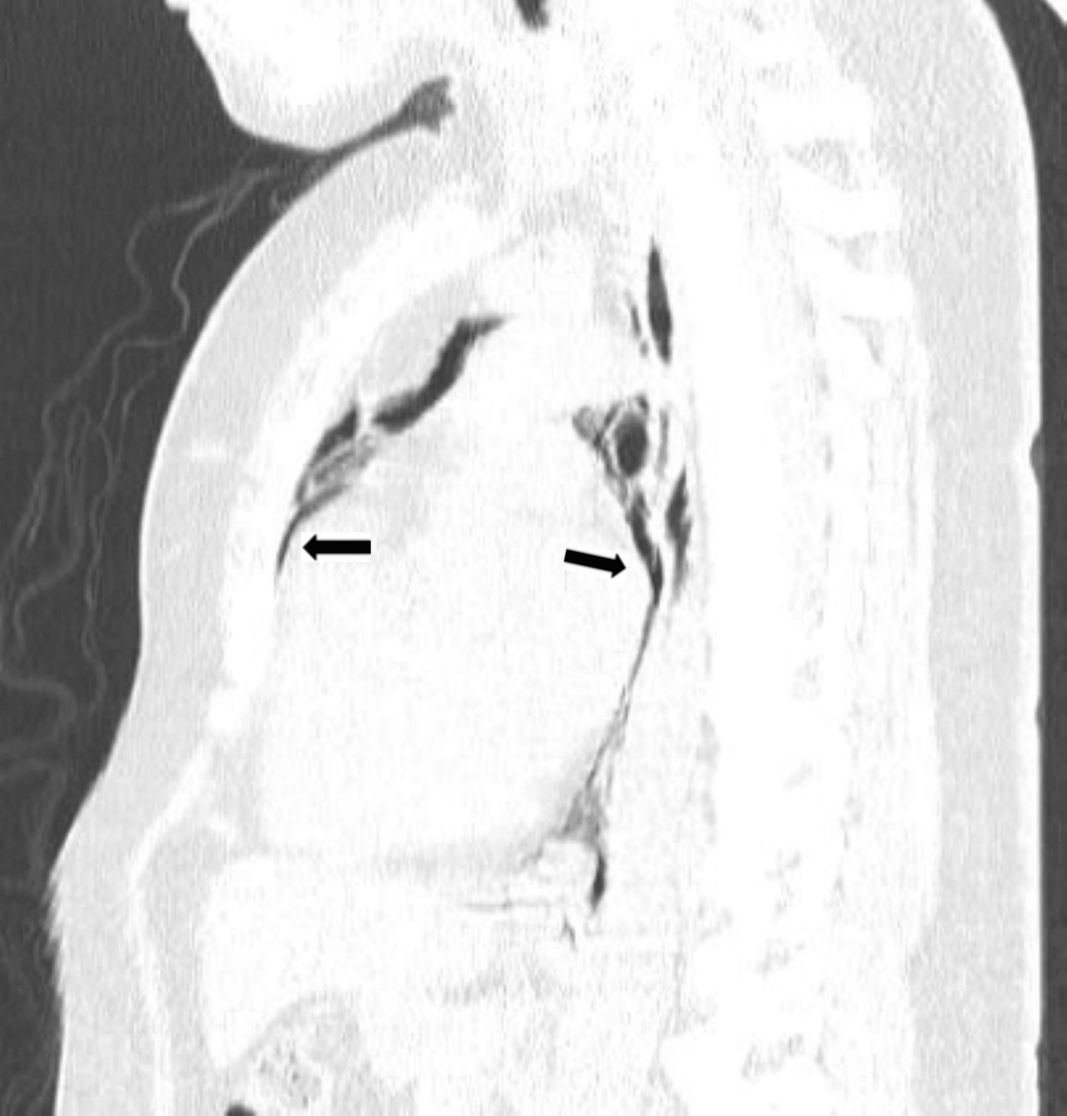
Sagittal chest CT scan showing moderate pneumomediastinum (arrows)

A chest CT scan revealed moderate pneumomediastinum associated with bilateral interstitial emphysema dissecting the peribronchovascular sheaths due to the Macklin effect, with no direct or indirect signs of pulmonary embolism (Figures 2-4). A contrast-enhanced CT scan was attempted but was unsuccessful; nonetheless, a proximal pulmonary embolism was effectively excluded.

The patient was managed with close clinical monitoring, low-flow oxygen therapy, and strict bed rest. Her condition improved rapidly, with resolution of retrosternal pain and dyspnea within 48 hours. She was discharged after a five-day hospital stay. A follow-up chest X-ray performed one week later showed no abnormalities.

## Discussion

Spontaneous pneumomediastinum in the postpartum period is defined as the presence of air within the mediastinum in the absence of any notable traumatic or iatrogenic cause [1]. It is a rare and generally benign condition, typically occurring in young women, with an estimated incidence ranging from 1 in 2000 to 1 in 100,000 deliveries [1,4,5].

The pathophysiology of SPM was first described by Macklin and Macklin in 1944 [3], and then by Maunder et al. in 1984 [6]. Pneumomediastinum may result from mediastinal infection with gas-producing bacteria, tracheobronchial or esophageal rupture, or alveolar rupture [1]. The last mechanism underlies spontaneous pneumomediastinum via the Macklin effect, which can occur following prolonged effort against a closed glottis, such as the Valsalva maneuver during labor. Increased intra-alveolar pressure leads to alveolar rupture, allowing air to dissect along the peribronchovascular sheaths and into the mediastinum, resulting in spontaneous pneumomediastinum [3,7].

In our case, prolonged labor and sustained expulsive efforts likely contributed to the development of SPM, presenting with retrosternal chest pain, mild hemoptysis, and sudden-onset dyspnea in the postpartum period. Our patient did not exhibit subcutaneous emphysema, and Hamman’s sign was present, consistent with previous reports. In the literature, the most common clinical symptom is retrosternal chest pain. Neck pain, sudden-onset dyspnea, and persistent cough are reported in approximately half of the cases. The most frequently observed clinical sign is subcutaneous emphysema, particularly in the cervical region. It is also important to note Hamman's auscultatory sign, which is a pathognomonic sign of pneumomediastinum [1,8,9].

It is therefore essential to rule out other life-threatening differential diagnoses, including pulmonary embolism, aortic dissection, spontaneous pneumothorax, and cardiac tamponade [4]. A chest X-ray can support the diagnosis, typically demonstrating a thin radiolucent line outlining the cardiac silhouette and subcutaneous hyperclarities in the cervical region, although false-negative results may occur. A CT scan is particularly valuable in identifying the Macklin effect, with air dissecting along the peribronchovascular sheaths [10].

The natural evolution of postpartum pneumomediastinum is generally favorable, with spontaneous resolution of clinical signs within 48-96 hours. Management is typically conservative, including close clinical monitoring, vital sign assessment, pain control, oxygen therapy when indicated (particularly in cases of desaturation), and bed rest, accompanied by radiological follow-up during hospitalization. Recurrences are rare, and complications are exceptional [11,12]. In some cases, pneumomediastinum may unmask previously undiagnosed asthma, highlighting the importance of pulmonary function testing once symptoms have resolved [13].

Spontaneous pneumomediastinum in the postpartum period is a rare and generally benign condition that typically resolves spontaneously, with complications remaining exceptional. A thorough understanding of the clinical presentation, combined with accurate radiological interpretation, allows the diagnosis to be made without resorting to invasive or unnecessary examinations such as bronchoscopy or endoscopy.

It is therefore important to educate the obstetric team about the specificity of postpartum pneumomediastinum and to provide guidance on directed pushing techniques during labor. This approach, which is not used in routine practice, could reduce the risk of pneumomediastinum. Nevertheless, it is essential to remain vigilant for warning signs of tracheobronchial injury or esophageal rupture, as seen in Boerhaave's syndrome, particularly in the presence of fever, dysphagia, vomiting, or severe dyspnea. Such cases may require endoscopic confirmation and surgical management [14].

## Conclusions

Spontaneous postpartum pneumomediastinum due to the Macklin effect is a rare and generally benign condition resulting from alveolar rupture after prolonged labor. Increased intrathoracic pressure leads to air dissecting along the peribronchial sheaths and entering the mediastinum. The diagnosis can be made based on the patient’s history and clinical examination. Radiological imaging, particularly chest CT, confirms the diagnosis and helps exclude differential diagnoses and other potentially life-threatening conditions. Management is primarily supportive, with favorable clinical outcomes, and recurrences are uncommon. This case underscores the importance of clinician awareness and early recognition in postpartum women to prevent unnecessary invasive investigations and improve patient care.
